# Editorial: Biopolymer Thin Films and Coatings

**DOI:** 10.3389/fchem.2019.00736

**Published:** 2019-10-25

**Authors:** Stefan Spirk, Tiina Nypelö, Eero Kontturi

**Affiliations:** ^1^Institute of Paper, Pulp and Fiber Technology, Graz University of Technology, Graz, Austria; ^2^Department of Chemistry and Chemical Engineering, Chalmers University of Technology, Gothenburg, Sweden; ^3^Wallenberg Wood Science Center, Gothenburg, Sweden; ^4^Department of Bioproducts and Biosystems, Aalto University, Espoo, Finland

**Keywords:** thin films, biopolymer, cellulose, chitosan, lignin, coatings, proteins

The development of thin film technology has played a decisive role in the manufacturing of high-tech devices and products such as computers, mobile phones, solar cells, sensors, and displays. With polymer materials, synthetic polymers are still mostly applied while the use of bio-based materials for thin coatings and films is still at an early stage. Of these, particularly thin films of natural polysaccharides have been at the forefront of the research scene for the past 10–15 years. Although the main problems in film preparation, such as sparing solubility, have been largely overcome, many issues affecting the film performance are still not fully understood. One of the pressing issues is the influence of water on the film structure and behavior, given the special relationship that many biopolymers share with water. Other open questions include the stability and the general structural order of the films. In this special issue, we have assembled the frontiers of biopolymer ultrathin films: topical studies on water interactions, protein adsorption, and film structure among many others. The materials choices in the thin films include cellulose, cellulose derivatives, lignin, alginate, and chitosan.

The special issue contains two review and 10 original research articles. The reviews yield insight into general materials perspectives (Kontturi and Spirk) as well as to the current state of ultrathin cellulose films to act as a biointerface (Raghuwanshi and Garnier). These are important emerging areas particularly with polysaccharide thin films where the research has generally been focused on their use as model systems (model surfaces). The 10 research articles, on the other hand, focus on different, mainly fundamental and materials-related aspects, such as composition, morphology and accessibility, crosslinking and interaction with biomolecules. The contribution of Kargl et al. presents affinity of proteins on cellulose, and other surfaces, and contributes to the quest for identifying the reasons behind selectivity of protein attachment on surfaces. Quantification of protein adsorption on surfaces in aqueous media is limited by the physics of the analytical techniques but also of data analysis. This contribution among others, utilizes a combination of analytical methods to cover the limitations of the individual techniques and models.

Czibula et al. investigate mixtures of two biopolymers in a film. They present film formation from cellulose derivate blends, leading to control of hydrophobic and hydrophilic domains, combined with surface roughness and structure periodicity. There is something curious about proteins adsorbing on textured surfaces that lead often to an unexpected increase in the adsorbed amount at certain compositions, as seen in previous accounts. The contribution by Czibula contributed to this topic by adding friction force morphology as a tool to screen interactions besides conventional tools such as QCM-D and SPR. Nau et al., on the other hand, focused on film formation by cross-linking hydroxypropyl cellulose that had been modified with fatty acid esters. Cross-linking and simultaneous binding on the surface was realized with a photo-induced radical cross-linker and the film properties were carefully and comprehensively monitored.

Besides macromolecular interaction, adsorption of small molecules (other than water) has been probed relatively little with cellulose thin films. Henögl et al. looked at the deposition of small molecules on cellulose surfaces with regard to their interaction capacity. They used as model compounds n-decane and deuterated methanol and found that although both molecules adsorb, the interaction of methanol with the cellulose surfaces is stronger, and supported the observation with thermodynamic data. On the other hand, the work by Sampl et al. looked more on the order and density of such cellulose thin films. The paper reveals that there are different areas in the films that feature lower density, i.e., at the air-film interface and at the film-substrate interface. Maver et al. also look into the structural feature of films, specifically hydrophilic multi-layered thin films. They further apply them as a model drug-carrier and time-dependent release system aiming for new functional and active 3D materials to be used in regenerative medicine.

Hua et al. had a more pragmatic approach to coat hydrophobic films on down-to-earth substrates like glass or Kraft pulp sheets, resulting in increased surface hydrophobicity of these materials. The lignin coating was performed using microparticles whose formation was made feasible by esterification of lignin hydroxyl groups. Likewise, Chemin et al. had their eye on utilizing thin films for genuine applications. In their work, hybrid films of cellulose nanocrystals and Gibbsite nanoplatelet films were constructed by layer-by-layer technique onto substrates in packaging applications, such as cardboard. The resulting materials exhibited improved oxygen barrier properties. Electrostatic interaction was also utilized in a different fashion by Zou et al. who used chitosan to attach as an ultrathin biofilm on negatively charged colloidal lignin nanoparticles. Such nanoparticles were especially suited for stabilizing oil-in-water Pickering emulsions for, e.g., food applications. Work of Zieglowski et al. also utilizes lignin. They provide insight in reactivity and isocyanate structure linked to Kraft lignin on polyurethane formation. Polyurethanes are an important material for coatings and use of lignin enables incorporation of the biomacromolecule into a highervalue polymer material. The rational choice of the isocyanate for pre-functionalization of lignin assists to prepare materials with better mechanical performance.

The contributions in this special issue of Biopolymer Thin Films and Coatings highlight that mastering the fundamentals of film formation and characterization already enables to reach toward utilization. The applications covered in this special issue, supported by the state-of-the-art analysis, provide a dissection of the status of the biopolymer thin film and coating field. We hope that the issue will inspires the readers to further contribute to this exciting field of fundamental and applied endeavors.

## Author Contributions

All authors listed have made a substantial, direct and intellectual contribution to the work, and approved it for publication.

### Conflict of Interest

The authors declare that the research was conducted in the absence of any commercial or financial relationships that could be construed as a potential conflict of interest.

